# Notes from Private Practice

**Published:** 1878-05

**Authors:** W. L. Ransom

**Affiliations:** Roscoe


### NOTES FROM PRIVATE PRACTICE.

*Cancer of Stomach and Liver.*

Wm. C. J., aged 47, merchant, a resident of Nebraska, began to experience severe gastric pain in March, 1877. The attacks of pain recurred at irregular intervals, varying from two or three days to as many weeks; they were somewhat mitigated by moderate pressure, as in leaning over the counter; they were accompanied by considerable nausea and occasionally by vomiting.

No examination being made at the time, the disease was supposed to proceed from gastric irritation or dyspepsia; and for several months was treated in accordance with that supposition.

No improvement taking place, it was thought expedient that he should return to his old home in Illinois.

When first examined by me (Sept. 13, 1877) he complained most of distress from abdominal distension occasioned by ascites. The appetite was poor, but he had no nausea; pulse 80 to 84 per minute and weak. He was emaciated and feeble but able to walk about. Bowels regular (occasionally loose), urine natural in appearance, though inclined to be scanty.

His complexion was of that peculiar saffron hue common in patients where the cancerous diathesis obtains; how much this hue was dependent upon that diathesi in our patients, and how much upon obstruction of function in the liver, we may not now say. His disposition was cheerful and hopeful, expecting a speedy recovery, despite the unfavorable diagnosis — cancerous tumor in the vicinity of the stomach.

Palpation of abdomen revealed a nodular prominence about the size and shape of a walnut, three inches inferior to the ensiform extremity of the sternum; from this eminence a hard, irregular surface extended toward the left side, disappearing under the adjacent costal cartilage. Inferior to the left thorax, and on a line with the corresponding nipple the irregular surface could be distinctly felt.

Deep pressure revealed a smooth surface in the right hypochondriac region.

The abdomen was considerably distended by fluids which seemed to interfere with his appetite, and also prevented an extended dorsal decubitus.

The ankles and feet were very œdematous, pitting on pressure.

The treatment consisted at first in painting the integument over the tumor with the tincture of iodine (which was continued throughout this illness, whenever the condition of the skin would permit). Considerable relief seemed to ensue from these applications. Tonic powders of cinchona, lacto-peptine, bismuth, and lactate of iron, were also exhibited.

Sept. 17.—The ascites still continuing, diuretics were administered, and fomentations of digitalis leaves applied over the abdomen.

Sept. 22d.—Distress from abdominal distension still persisting, it was thought expedient to remove the fluid by aspiration. By means of Mathew's apparatus, about six pounds of amber-colored serum were removed, the puncture being made midway between the umbilicus and the symphysis pubis.

This operation afforded considerable relief to the patient, who was thus enabled to assume the dorsal decubitus, which had been impracticable for six months on account of the pressure.

Sept. 24th.—Appetite and digestion much improved.

Sept. 28th.—Is troubled with pyrosis, nausea, and subsequently by vomiting ; these symptoms are much relieved when he lies in such a manner that the tumor does not press directly upon the stomach. No signs of reaccumulation of fluids in abdomen. As the patient seems much debilitated from weakness of the stomach and want of proper nutrition, rectal alimentation was resorted to by administering eggs, whisky, and beef tea, which seemed to revive the patient, materially improving his strength and general appearance.

Emaciation and absence of ascitic fluids, render it easy to examine the tumor, which has become less painful, and is easily moved in various directions, even by the patient himself. Numerous fissures could be readily detected traversing the tumor in various directions—the *sulci* between masses of cancerous enlargements.

Oct. 1.—Vomited matter slightly tinged with bile. This act prostrated him exceedingly, and recurred every two or three days, rendering the patient still weaker.

Oct. 8.—Nourishing enemata fail—returning undigested. An excellent substitute was prepared in the following way: Two or three ounces of raw beef were thoroughly triturated in a mortar, slowly adding a little water, till nearly all the red portion of the meat had been dissolved out, leaving the white fibrous portion, which was separated from the former by straining through muslin.

To the solution thus prepared, pepsin was added; and the whole gently warmed and thrown into the rectum.

The beneficial effects of this mode of nourishment became manifest in less than an hour, proving to be very refreshing and strengthening to the patient. These injections were repeated every three or four hours.

On the 10th, the bowels moved in such a way as to exhaust him exceedingly. As he exhibited symptoms of rapid failure, and in order that he might survive until the arrival of his wife, it was deemed advisable to prolong life, if possible, by means of transfusion of blood.

Accordingly, on the 12th, Mr. O. E. Bowers, a strong, healthy soldier, who had upon two previous occasions furnished blood for a similar purpose, presented himself, willing to provide the requisite amount of blood.

With the assistance of Dr. C. T. Fenn, of Chicago, the operation was performed, after the immediate or direct method, as follows: The apparatus was first completely filled with warm water; an incision was then made into the right basilic vein of the donor, cording the arm above the point, a corresponding opening was made in the right basilic vein of the patient, without cording the arm. The transfusion apparatus was then adjusted by inserting the canulæ filled with water into the veins of donor and patient respectively. The rubber tube and bulb (filled with water) were adjusted to the canulæ (care being taken that air should not enter any portion of the apparatus). Compressing the donor's end of the tube, the water in the apparatus was forced into the patient's vein; compressing the patient's end of the tube, and allowing the bulb to expand, blood from the donor was thus drawn into the instrument, and thrown into the patient's vein in the manner described above.

In this manner, three and one-half ounces of fresh blood were transfused, when we were obliged to stop the operation in consequence of some obstruction in the tubes.

The patient expressed himself as feeling stronger, better able to breathe, and to have acquired a new lease of life. The pulse, which before the operation, was very weak, subsequently became strong, full, and regular.

This sense of strength, however, soon began to die away—the pulse becoming almost imperceptible, until about ten hours after the operation, when he gradually revived again, and with unusual strength, proceeded to transact business affairs, greeting and conversing quite naturally with his wife and children, who had recently arrived, even then expressing great hopes of eventual recovery.

In two or three hours the bowels moved again, prostrating him exceedingly, and at 9 a. m. on the 13th, he vomited with great effort a very tenaceous, greenish matter, containing purulent masses. After this he sank gradually, without consciousness, dying at 3:10 p. m.

*Autopsy*, nineteen hours after death. No marked *rigor mortis ;* great emaciation. Palpation and percussion revealed a tumor occupying the space already described, dullness on the right side, commencing at the sixth rib, on the left at the eighth.

On opening the abdomen, the tumor was seen to be an enlarged liver, extending over the epigastric region into the left hypochondriac. It was filled with a large number of white scirrhous masses, varying from the size of a grape seed to that of a large egg. Many of the larger ones projecting from the surface of the liver, presented points of commencing disintegration. One of these, immediately inferior to the ensiform cartilage, was quite prominent—easily examined during life. The intestines were empty, and at various points adherent to the inferior surface of the liver.

The weight of the latter organ was eight pounds nine ounces.

A large portion of the stomach, involving its pyloric extremity, was firmly adherent to the liver, the pancreas and various adjacent tissues, including those investing the abdominal aorta and spinal column, the media of this union being tough irregular bands of cancerous tissue, firmly binding into one ragged mass, the organs and membranes mentioned. Considerable force was required to tear them asunder.

The tissue of the pylorus was transformed into a hard gristly tube, one and a half inches in length, through which the index finger could be thrust.

Right heart œdematous, valves normal; both ventricles contained large shreds or clots of white fibrin; spleen and kidneys, normal.

This case presents two or three points of interest:

*The use of the aspirator* in fluid accumulations in the abdomen. Attempts had been made to cause the removal of the fluid by exhibiting diuretics; we were partially successful in the use of apocynum cannabinum, but it was evident that the waning strength would not permit the long continued employment of this mode of treatment. Hence the necessity for this operation.

*The utility of rectal alimentation*, when functional or structural derangement of any portion of the alimentary canal exists, is fully demonstrated in this case. Two other cases have come under my observation, in both of which the prospect of recovery seemed very forbidding, owing to the extensive chronic gastritis, in consequence of which the system was insufficiently nourished. But by judicious preparations and use of raw beef, as above described, the diseased organ was allowed to rest, recovery ensuing within a few weeks, complete restoration of health and strength in the course of a few months.

The first of these cases was that of a farmer, who had induced a severe type of gastritis from excessive use of alcoholic drinks while residing near Rockford. He was sent to his home on the farm, incurable. When seen by me, on July 3d, 1875, the patient had been able for four or five months, to retain in the stomach but very little nourishment; even water, at times, excited emesis. He was very much emaciated. Giving only powders of cinchona, lactopeptine, and bismuth, by the stomach, nourishment was administered by the rectum. Symptoms of improvement gradually appeared, and in a few weeks hopes of recovery were entertained. Continuing the above treatment persistently, the patient was discharged cured, Nov. 1st, 1875.

*Transfusion.* This operation, although incapable of serving as a means of recovery, prolonged existence so that the patient was enabled to adjust his affairs, which evidently could not have been accomplished otherwise. With due caution, the operation may be performed very easily, quickly, and safely.

I shall not hesitate to employ transfusion by the "direct method," whenever sufficiently indicated, as when death is imminent from anæmia or malnutrition.

W. L. Ransom, M. D.

Roscoe, March 23d, 1878.

---

A Remedy for the Eruption Produced by Poison Oak, Ivy and Sumach.—Dr. S. A. Brown, U. S. N., Mare Island, Cal., believes that he has found a specific for the eruption caused by contact with poison oak, etc. He writes: "This specific is *bromine.* I have used it with the same unvarying success in at least forty cases. The eruption never extends after the first thorough application, and it promptly begins to diminish. Within twenty-four hours, if the application be persisted in, the patient is entirely cured. I use the bromine dissolved in olive oil, in cosmoline, or in glycerine. The application with glycerine is painful, and, I think, possesses no advantage to compensate for the irritation. The strength of the solution is ten to twenty drops of bromine to the ounce of oil, used by rubbing gently on the affected part three or four times a day, and especially on going to bed at night. The bromine is so volatile that the solution should be renewed within 24 hours of its preparation.—*N. Y. Med. Record.*

